# Suppression of microRNA-31 increases sensitivity to 5-FU at an early stage, and affects cell migration and invasion in HCT-116 colon cancer cells

**DOI:** 10.1186/1471-2407-10-616

**Published:** 2010-11-09

**Authors:** Chao-Jie Wang, Johannes Stratmann, Zong-Guang Zhou, Xiao-Feng Sun

**Affiliations:** 1 Division of Oncology, Department of Clinical and Experiment Medicine, University of Linköping, Linköping, Sweden; 2 Department of Gastrointestinal Surgery, West China Hospital, Sichuan University, Chengdu, PR China

## Abstract

**Background:**

MicroRNAs (miRNAs) are endogenously expressed noncoding RNAs with important biological and pathological functions. Although several studies have shown that microRNA-31 (miR-31) is obviously up-regulated in colorectal cancer (CRC), there is no study on the functional roles of miR-31 in CRC.

**Methods:**

Anti-miR™ miRNA 31 inhibitor (anti-miR-31) is a sequence-specific and chemically modified oligonucleotide to specifically target and knockdown miR-31 molecule. The effect of anti-miR-31 transfection was investigated by real-time PCR. HCT-116^p53+/+ ^and HCT-116^p53-/-^colon cancer cells were treated by anti-miR-31 with or without 5-fluorouracil (5-FU), cell proliferation was determined by MTT assay; apoptosis was detected by DAPI staining; cell cycle was evaluated by flow cytometry; colony formation, migration and invasion assays were performed to investigate the effect of suppression of miR-31 on the cell lines.

**Results:**

Real-time PCR results showed that anti-miR-31 was efficiently introduced into the cells and reduced miR-31 levels to 44.1% in HCT-116^p53+/+ ^and 67.8% in HCT-116^p53-/-^cell line (*p *= 0.042 and 0.046). MTT results showed that anti-miR-31 alone had no effect on the proliferation of HCT-116^p53+/+ ^or HCT-116^p53-/-^. However, when combined with 5-FU, anti-miR-31 inhibited the proliferation of the two cell lines as early as 24 h after exposure to 5-FU (*p *= 0.038 and 0.044). Suppression of miR-31 caused a reduction of the migratory cells by nearly 50% compared with the negative control in both HCT-116^p53+/+ ^and HCT-116^p53-/-^(*p *= 0.040 and 0.001). The invasive ability of the cells were increased by 8-fold in HCT-116^p53+/+ ^and 2-fold in HCT-116^p53-/- ^(*p *= 0.045 and 0.009). Suppression of miR-31 had no effect on cell cycle and colony formation (*p *> 0.05).

**Conclusions:**

Suppression of miR-31 increases sensitivity to 5-FU at an early stage, and affects cell migration and invasion in HCT-116 colon cancer cells.

## Background

MicroRNAs (miRNAs) are endogenous non-coding, single-stranded RNAs of ~22 nucleotides which function as post-transcriptional gene regulators [1]. More recently, aberrant miRNAs expression profiles have emerged as potential indicators of cancer development and progression, acting as major regulators of genes involved in oncogenesis and tumor suppression [2]. MiRNAs regulate their targets depending on the degree of complementarity between miRNAs and targets, and result in the degradation and/or translation inhibition of target mRNAs [3]. These studies using bioinformatics algorithms have revealed that a single miRNA might bind to as many as 200 gene targets and that targets can be diverse in their function; they include transcription factors, secreted factors, receptors and transporters. So, miRNAs potentially control the expression of about one-third of human mRNAs [4]. Thus, miRNAs add a whole new layer of complexity by which large numbers of genes and their biological processes can be broadly regulated.

MicroRNA-31 (miR-31), located on chromosome 9p21.3, was first identified in HeLa cells [5]. More and more evidence shows that miR-31 has different expression pattern in different cancers: it is up-regulated in colorectal cancer (CRC) [6-10], head and neck squamous cell carcinoma (HNSCC) [11], hepatocellular carcinoma [12], squamous cell carcinoma of tongue [13], and lung cancer [14]; but it is down-regulated in invasive urothelial carcinoma of the bladder [2], prostate cancer [15], gastric cancer [16], breast cancer [17], and serous ovarian cancer [18]. Although there is growing evidence that miR-31 level varys among cancer types, functional roles for miR-31 have yet to be defined. Recent studies have shown that miR-31 expression is specifically attenuated in metastatic breast cancer cell lines and miR-31 inhibits breast cancer metastasis by targeting multiple genes [17]; miR-31 is underexpressed in serous ovarian carcinomas, and in a number of serous cancer cell lines with a dysfunctional p53 pathway (OVCAR8, OVCA433, and SKOV3), miR-31 overexpression inhibits proliferation and induces apoptosis; however, in other lines with functional p53 (HEY and OVSAYO), miR-31 has no effect [18]. MiR-31 is up-regulated in HNSCC, ectopic expression of miR-31 increases the oncogenic potential of HNSCC cells under normoxic conditions in cell culture or tumor xenografts. Conversely, blocking miR-31 expression reduces the growth of tumor xenografts [11].

Although several studies have shown that miR-31 is up-regulated in CRC, there is no study on the functional roles of miR-31 in CRC, and little is known about the role of miR-31 in modulating the tumor cell response to 5-fluorouracil (5-FU). The anti-miRNA inhibitor is a sequence-specific and chemically modified oligonucleotide to specifically target and knockdown miRNA molecule, and has been applied to investigate miRNA functions in several studies [19,20]. In the present study, we applied anti-miR™ miRNA 31 inhibitor (anti-miR-31) to knockdown miR-31 molecule in the HCT-116^p53+/+ ^and HCT-116^p53-/-^colon cancer cells, and evaluated the role of miR-31 in the response of HCT-116 to 5-FU. The results suggest that suppression of miR-31 increases sensitivity to 5-FU at an early stage, and affects cell migration and invasion in HCT-116 colon cancer cells.

## Methods

### Cell culture and transient transfection

Human colon cancer cell lines HCT-116 with wild type p53 (HCT-116^p53+/+^) or mutated p53 (HCT-116^p53-/-^) used in this study were a kind gift from Dr. Bert Vogelstein (Johns Hopkins University, Baltimore, MD). Two wild type p53 alleles in HCT-116 ^p53-/- ^have been targeted by homologous recombination, resulting in a mutated p53 with a 40 amino acid truncation, and the HCT-116^p53-/- ^cells do not express detectable wild type p53 [21]. The two cell lines were cultured in McCoy's 5A medium (Sigma-Aldrich, Stockholm, Sweden) supplemented with 10% FBS (Invitrogen, Carlsbad, CA), 1% PEST (Invitrogen) and 1.5 mM L-glutamine (Invitrogen).

Anti-miR™ miRNA 31 inhibitor (anti-miR-31) (Applied Biosystems, Foster City, CA) or Anti-miR™ negative control (Applied Biosystems) were transfected at a final concentration of 100 nM using the siPORT™ NeoFX™ Transfection Agent (Applied Biosystems) according to the manufacture's protocol.

### RNA extraction and quantitative reverse transcription-PCR analysis

Total RNA, inclusive of the small RNA fraction, was extracted from cells using mirVana™ miRNA Isolation Kit (Ambion Inc., Austin, TX) according to the manufacture's protocol. The RNA concentration was quantified using a ND-100 Spectrophotometer (Nanodrop technologies, Wilmington, DE). TaqMan MicroRNA assays (Applied Biosystems) were used to quantify miR-31 expression, and RNU6B (Applied Biosystems) was used as the endogenous control.

Gene-specific reverse transcription (RT) for miR-31 and RUN6B using about 200 ng of purified total RNA, 0.15 μL of 100 mM dNTPs (with dTTP), 1.5 μL 50 u/μL MultiScribe Reverse Transcriptase, 1.5 μL 10 × RT Buffer, 0.188 μL RNase Inhibitor, 3.0 μL 5 × TaqMan microRNA RT Primer and 4.162 μL nuclease free water. Fifteen microliter reaction was incubated for 30 min at 16°C, 30 min at 42°C, and 5 min at 85°C to inactivate reverse transcriptase. Real-time PCR reaction, including 1.33 μL of RT product, 7.67 μL nuclease free water, 10 μL TaqMan 2 × Universal PCR master mix (Applied Biosystems) and 1 μL TaqMan microRNA Assay containing PCR primers and TaqMan probes, was run in triplicate on the ABI Prism 7500 HT Sequence Detection System (Applied Biosystems) at 95°C for 10 min followed by 40 cycles at 95°C for 15s and 60°C for 1 min. MiR-31 expression change was normalized to RNU6B, and calculated using the 2^-ΔΔCt ^method. Each test was repeated in triplicate.

### MTT assay

Cell proliferation was measured by the 3-(4, 5-Dimethylthiazol-2-yl)-2, 5-diphenyltetrazolium bromide (MTT) (Sigma-Aldrich) assay. 5-FU (Sigma-Aldrich) stock solution of 80 mM was prepared in dimethyl sulfoxide (DMSO) (Sigma-Aldrich). For the 5-FU dose-response curve, cells were plated at a density of 4.8 × 10^3 ^cells/well, and incubated for 24 h. 5-FU was added to the wells at a final concentration of 2, 4, 6, 8 and 10 μM, and continued to incubate for another 48 h. The final DMSO concentration was always less than 0.1%. Then, 20 μL of 5 mg/mL solution of MTT in PBS was added to each well. The plates were incubated for another 4 h at 37°C. The precipitate was solubilized in 100% DMSO 150 μL/well, and shaken for 10 min. Absorbance of each well was measured on a microplate reader (Anthos ht III, Anthos Labtec Instruments GmbH, Wals, Austria) at a wave length of 490 nm.

To investigate the effect of miR-31 on the response of HCT-116 cells to 5-FU, cells were transfected with 100 nM of either anti-miR-31 or Anti-miR™ negative control in 96-well plates at a density of 2 × 10^3 ^cells/well, and incubated for 24 h. 5-FU was added to the well at a final concentration of 8 μM. At the indicated time points, cell viability was tested by MTT assay. Each test was repeated in triplicate.

### DAPI staining

At the given time point, the cells in culture were centrifuged to spin the apoptotic cells to the bottom of the plate and were trypsinized with TrypLE (Invitrogen). The pellet was resuspended in PBS and cells were centrifuged on to a glass slide with a Shandon Cytospin^® ^2 Cytocentrifuge (Thermo ScientiWc, Waltham, MA). The cells on the slides were fixed with buffered formalin for 10 min, washed in ice cold PBS for 10 min and then stained/mounted with VECTASHIELD^® ^HardSet™ Mounting Medium with diamidino-2-phenylindole (DAPI) (Vector Laboratories, Burlingame, CA). The cells were morphologically examined by two independent investigators in a fluorescence microscope with UV light and judged as either apoptotic or non-apoptotic (areas of about 200 cells were counted). Each test was repeated in triplicate.

### Cell cycle analysis

Cells were transfected with 100 nM of either anti-miR-31 or negative control in 24-well plates at a density of 2.7 × 10^4 ^cells/well, and incubated for 48 h. Cells were trypsinized and prepared for cell cycle analysis using the Vindelöv protocol with propidium iodine (PI) for DNA staining [22]. Cell cycle analysis was performed using a FACScan (Becton-Dickinsson, San José, CA). The data were analyzed and calculated by ModFit LT for Mac V 3.1 (Verity Software House, Inc., Topsham, ME). Each test was repeated in triplicate.

### Colony formation assay

Log-phase cells were transfected with 100 nM of either anti-miR-31 or negative control, seeded in the 6-well plates with 2 ml medium (500 cells/well), and incubated at 37°C in a humidified incubator. Plates were initially examined microscopically to confirm that only single cells without clumps had been plated. Five days after plating, colonies were fixed with 4% formaldehyde and stained with 5% Giemsa in 95% ethanol. Colonies were visualized with Leica microscope (Leica Microsysytems wetzlar GmbH, Germany) and pictures were taken with Olympus SC20 camera and soft imaging system (Olympus soft imaging solution GmbH, Germany). The colonies with a diameter of > 0.3 mm were counted under light microscope at 4 × magnification, and the colony forming efficiency (CFE) was determined by the percentage of colonies per well. Each test was repeated in triplicate.

### Cell migration and invasion assay

Cells were transfected with 100 nM of either anti-miR-31 or negative control, and incubated for 24 h, then the cells were starved by incubating for 12 h in serum-free medium. For the migration assay, 4 × 10^4 ^cells were resuspended in 300 μL serum-free medium and added into the upper chamber of the QCM™ 24 Well Colorimetric Cell Migration Assay (Millipore Corporation, Billerica, MA); For the invasion assay, 8 × 10^4 ^cells in 300 μL serum-free medium were added into the upper chamber of the Cell Invasion Assay Kit (Millipore Corporation). 500 μL of 10% FBS medium was added to the lower chamber. Thereafter the cells were incubated 16 h for migration assay and 48 h for invasion assay. Then the cells/media were carefully removed from the top side of the insert by pipetting out the remaining cell suspension, a cotton-tipped swab was used to gently remove non-migratory or non-invasive cells from the interior of the insert. The insert was stained and the images from 5 representative fields of each membrane were taken. The amount of migratory and invasive cells in the lower chamber was counted. Each test was repeated three or four times.

### Statistical analysis

All data were expressed as the mean ± SEM from at least three independent experiments. Student's *t*-test for two groups or one-way analysis of variance (ANOVA) and post hoc multiple comparisons (LSD test) for three or four groups were performed to evaluate the statistical significance using the SPSS 11.5 statistical software package (SPSS, Inc., Chicago, IL). Tests were two-sided, and *p *< 0.05 was considered as statistically significant.

## Results

### Sensitivity of HCT-116 cells to 5-FU

**Figure 1:**
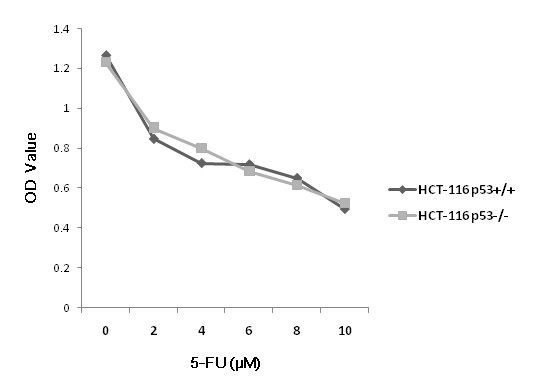
**5-FU induced cytotoxicity in HCT-116^p53+/+ ^and HCT-116^p53-/- ^cell lines in a dose-dependent manner at 48 h (*p *< 0.001)**.

We initially evaluated 5-FU cytotoxicity in both HCT-116^p53+/+ ^and HCT-116^p53-/- ^cell lines. Plasma concentration of 5-FU was more than 5 μM in patients subjected to continuous drug infusion at a constant rate of 450-966 mg/m^2^/day [23,24]. Borralho et al. used 8 μM 5-FU to treat HCT-116, and regarded it as a clinically relevant concentration [25]. Our result showed that the optical density (OD) value decreased in a dose-dependent manner after supplementation with 5-FU for 48 h (Figure 1, *p *< 0.001). Exposure to 0.1% DMSO solvent alone did not significantly alter the OD value (data not shown). So we used 8 μM 5-FU for the following experiments.

### Suppression of miR-31 increased sensitivity to 5-FU in HCT-116 cells

**Figure 2:**
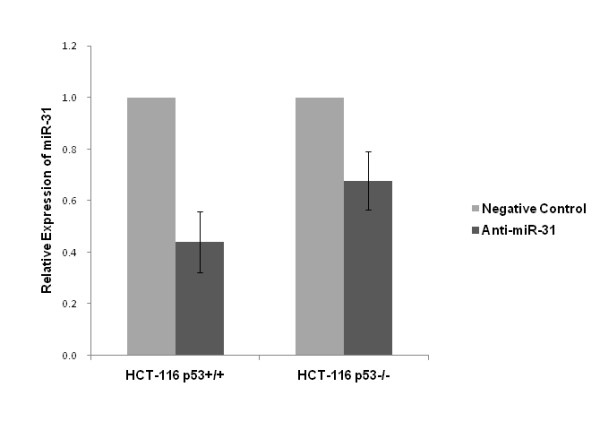
**Suppression of miR-31 expression by anti-miR-31 as detected by TaqMan real-time PCR**. Compared with negative control, miR-31 was reduced to 44.1% in HCT-116^p53+/+ ^(*p *= 0.042) and 67.8% in HCT-116^p53-/- ^(*p *= 0.046) cell line.

TaqMan real-time PCR revealed that anti-miR-31 transfection significantly reduced miR-31 levels to 44.1% in HCT-116^p53+/+ ^cell line (*p *= 0.042), and 67.8% in HCT-116^p53-/- ^(*p *= 0.046) compared with negative control (Figure 2), confirming that anti-miR-31 was efficiently introduced into the cells and knocked down miR-31.

**Figure 3:**
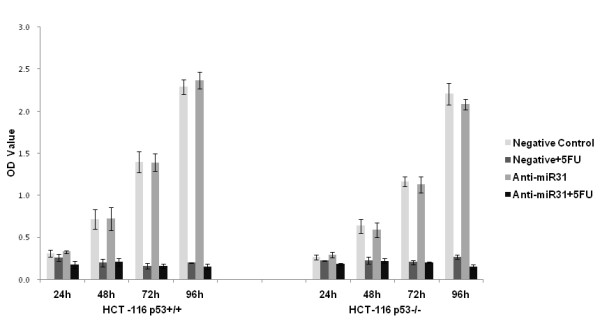
**MTT assay for cell viability at different time points**. Anti-miR-31 alone had no effect on the proliferation of either HCT-116^p53+/+ ^or HCT-116^p53-/- ^cell line (*p *> 0.05), but when combined with 5-FU, anti-miR-31 inhibited the proliferation of both cell lines as early as 24 h after exposure to 5-FU, i.e., the optical density (OD) value of anti-miR-31 + 5-FU group was the lowest among the four groups, and the difference between anti-miR-31 + 5-FU group and negative control or anti-miR-31 group was significant (*p *< 0.05).

MTT results showed that anti-miR-31 alone had no effect on the proliferation of either HCT-116^p53+/+ ^or HCT-116^p53-/- ^cell lines (*p *> 0.05). However, when combined with 5-FU, anti-miR-31 inhibited the proliferation of both cell lines as early as 24 h after exposure to 5-FU. As shown in Figure 3, at the 24 h timepoint, the OD value of the negative control and 5-FU combined treatment group was lower than either negative control or anti-miR-31 group, but the difference was not statistically significant (*p *= 0.382 and 0.221). When we combined anti-miR-31 with 5-FU, the OD value became the lowest among the four groups, and the difference between the anti-miR-31 plus 5-FU treatment group and the negative control or anti-miR-31 group was significant (*p *< 0.05). With prolongation of exposure (≥ 48 h), 5-FU exhibited a strong inhibition on the proliferation of both HCT-116^p53+/+ ^and HCT-116^p53-/- ^cell lines (*p *< 0.01), and the effect of anti-miR-31 on cell proliferation was covered by 5-FU.

**Figure 4:**
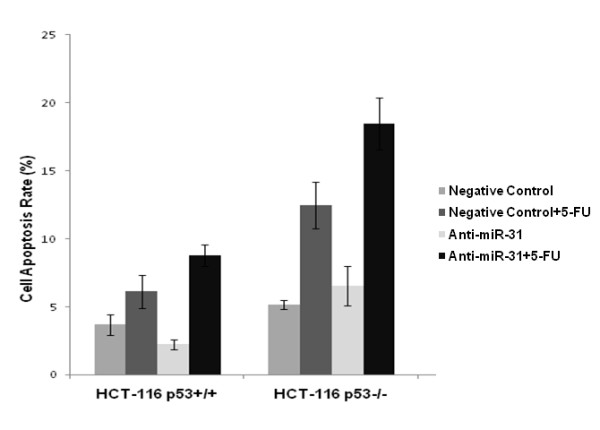
**DAPI staining for detecting the apoptotic cells at 24 h after exposure to 5-FU**. Apoptosis rate in anti-miR-31 + 5-FU group was the highest among the four different treatment groups of either cell line (8.8% ± 0.8% in HCT-116^p53+/+ ^and 18.4% ± 1.9% HCT-116^p53-/-^).

We used DAPI staining to detect the apoptotic cells after 24 h exposure to 5-FU. ANOVA analysis showed that apoptosis in different treatment groups were significantly different in both cell lines (p = 0.002 and 0.001), and, as shown in Figure 4, the apoptosis rate in the anti-miR-31 plus 5-FU group was the highest among the four different treatment groups of either cell lines (8.8% ± 0.8% in HCT-116^p53+/+ ^and 18.4% ± 1.9% HCT-116^p53-/-^).

Since 5-FU alone strongly inhibited the proliferation of HCT-116 cells *in vitro*, in the following experiments, we mainly focused on the anti-miR-31 and negative control groups in order to investigate miR-31 function.

### Suppression of miR-31 had no effects on cell cycle and colony formation in HCT-116 cells

**Figure 5:**
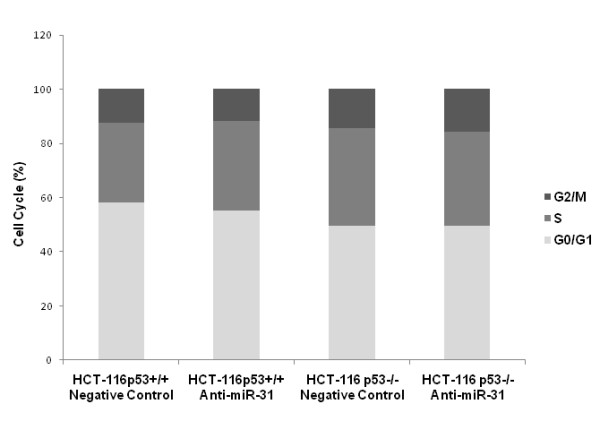
**Propidium iodine staining for detecting the cell cycle**. Suppression of miR-31 did not change cell cycle distribution either in HCT-116^p53+/+ ^or HCT-116^p53-/- ^cells.

**Figure 6:**
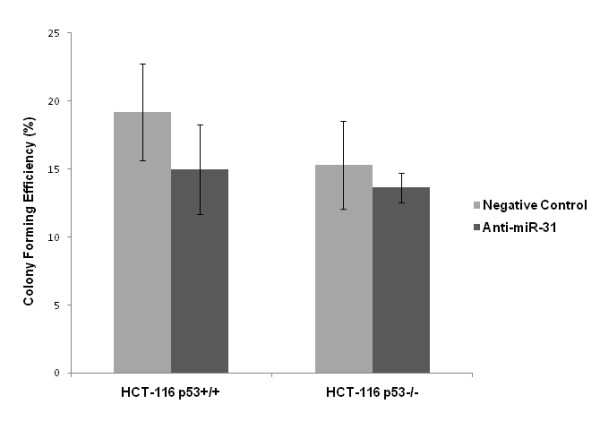
**Suppression of miR-31 had no effect on colony forming efficiency in either cell line (*p *> 0.05)**.

We examined the effect of anti-miR-31 on HCT-116 cell cycle and colony formation. Suppression of miR-31 did not change cell cycle distribution either in HCT-116^p53+/+ ^or HCT-116^p53-/- ^cells (Figure 5). Although suppression of miR-31 slightly reduced the colony forming efficiency, there was no statistical significance in both cell lines (Figure 6, *p *> 0.05).

### Suppression of miR-31 decreased migration but increased invasion in HCT-116 cells

**Figure 7:**
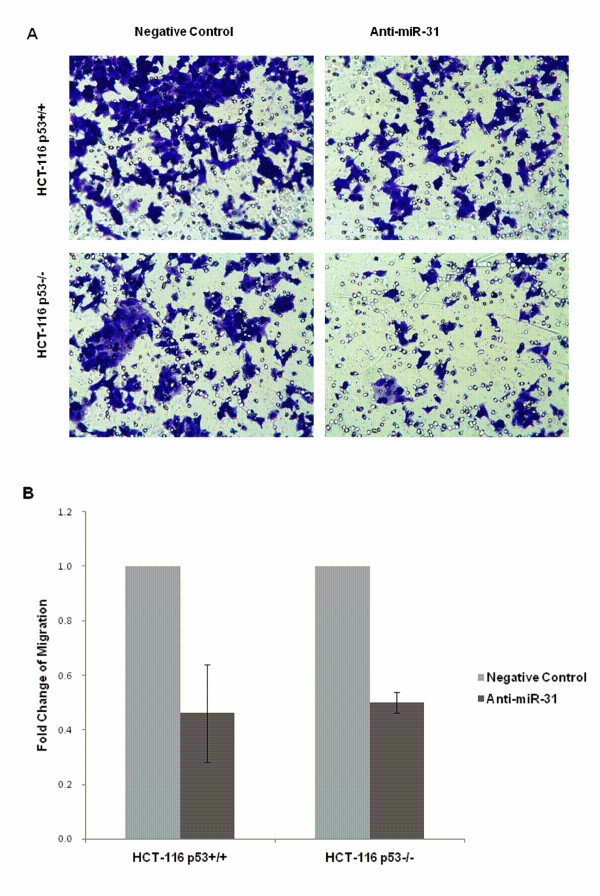
**Suppression of miR-31 decreased cell migration**. (A) Boyden chamber with 8 μm pore polycarbonate membrane was used for the migration assay. The chamber was stained and analyzed by photography, the stained cells were migratory cells in the lower chamber. (B) The migratroy cells decreased nearly 50% in both HCT-116^p53+/+ ^and HCT-116^p53-/- ^cells when they were normalized to negative control transfected cells (*p *= 0.040 and 0.001).

**Figure 8:**
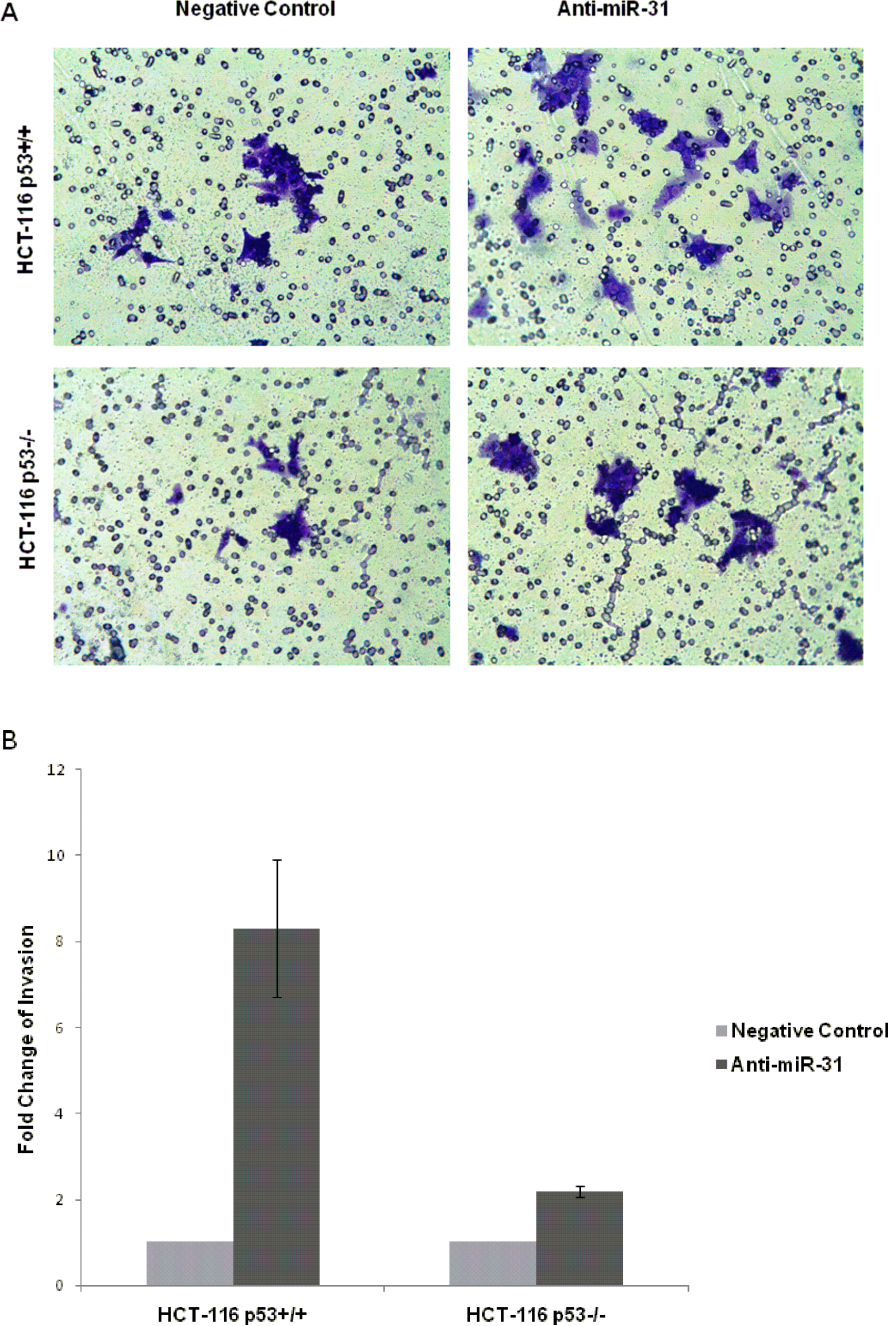
**Suppression of miR-31 increased cell invasion**. (A) Boyden chamber with 8 μm pore polycarbonate membrane coated with Matrigel was used for the invasion assay. The chamber was stained and analyzed by photography, the stained cells were invasive cells in the lower chamber. (B) The invasive cells increased by 8-fold in HCT-116^p53+/+ ^cell line (*p *= 0.045), and 2-fold in HCT-116^p53-/- ^cell line (*p *= 0.009).

We performed *in vitro *migration and invasion assays. Suppression of miR-31 significantly decreased cell migration ability in both HCT-116 cell lines. Although there were slight variations between different cell passages used in independent experiments, suppression of miR-31 persistently decreased migration by nearly 50% in both HCT-116^p53+/+ ^and HCT-116^p53-/- ^cells when the results were normalized to negative control transfected cells (*p *= 0.040 and 0.001, Figure 7). The invasive cells were increased by 8-fold in HCT-116^p53+/+ ^cell line, and 2-fold in HCT-116^p53-/- ^cell line (*p *= 0.045 and 0.009, Figure 8). The number of invasive cells were obviously less than migratory cells in both cell lines.

## Discussion

We were particularly interested in investigating the effect of miR-31 on the cellular response to 5-FU. Our results showed that although the suppression of miR-31 alone had no effect on cell proliferation, it did increase the sensitivity to 5-FU in HCT-116 cells as early as 24 h after exposure. At the same time point, combined treatment by 5-FU plus negative control did not significantly reduce the viability of the cells. With prolongation of exposure to 5-FU, both the combined groups, either 5-FU plus negative control or anti-miR-31, significantly reduced the cells viability. The apoptosis rate in the anti-miR-31 plus 5-FU group was the highest among the four different treated groups of either cell line, indicating that the anti-miR-31 plus 5-FU inhibited the cells proliferation partly through the apoptotic mechanism. However, the increase in the sensitivity of HCT-116 cells to 5-FU is quite modest and further studies are required to validate this phenomenon on more colon cancer cell lines, and even on other kinds of cancer cell lines.

Accumulating evidence suggests that miRNAs could be key players in regulation of tumor cell invasion and metastasis [26]. Our previous study showed that miR-31 expression was up-regulated in CRC compared to normal mucosa, and tumors which invaded adjacent tissues or organs had more miR-31 expression than those limited to the wall of the colon and rectum [6]. It was reasonable to speculate that miR-31 might affect the migration or invasion in CRC.

In the present study, the suppression of miR-31 caused a reduction of the migratory cells by nearly 50% compared with the negative control in both HCT-116^p53+/+ ^and HCT-116^p53-/- ^cell lines. This was in line with a provious study that blockage of miR-31 expression significantly decreased viability and migration in a HNSCC cell line [11]. Unexpectedly, our results showed that the invasive cells were increased by 8-fold in HCT-116^p53+/+ ^cell line, and 2-fold in HCT-116^p53-/- ^cell line. In HCT-116 cells, suppression of miR-31 decreased migration but increased invasion *in vitro*, this phenomenon might be partly explained by the fact that the migration assay detected the cell motility through an 8 μm pore polycarbonate membrane; however, the invasion assay not only evaluated cell motility but also the ability of tumor cells to invade through a basement membrane model (a reconstituted basement membrane matrix of proteins, including laminin and type IV collagen, derived from the Eangelbreth Holm-Swarm (EHS) mouse tumor [27]). So those cells that were capable of completing the entire process represented only a small subpopulation of the cells compared with the migration assay.

MiR-31 is down-regulated in serous ovarian cancer and miR-31 overexpression inhibits proliferation and induces apoptosis in a number of serous cancer cell lines with a dysfunctional p53 pathway. However, in other lines with functional p53, miR-31 has no effect [18]. On the other hand, overexpression of miR-31 suppresses metastasis in breast cancer cell lines, and does not affect proliferation *in vitro *[17]. In CRC, miR-31 was significantly up-regulated [6] and our present data showed that suppression of miR-31 increased the sensitivity of HCT-116 cells to 5-FU at an early stage, affected cell migration and invasion. Taken together, it seems that not only the expression but also the functions of miR-31 are cancer specific.

MiRNAs have been reported to be directly transactivated by p53; equally, p53 and components of its pathway have been shown to be targeted by miRNA thereby affecting p53 activities [28]. We asked whether miR-31 had a relationship with p53 in CRC. The HCT-116^p53+/+ ^and HCT-116^p53-/- ^cell lines are suitable to investigate the relationship between miRNAs and p53. Our results showed that the suppression of miR-31 had almost the same effects on cell cycle and colony formation. However, as for the cell invasion, it had a stronger effect on HCT-116^p53+/+ ^than HCT-116^p53-/- ^cell line, the apoptotic rate in HCT-116^p53+/+ ^was lower than that in HCT-116^p53-/- ^cell line, suggesting miR-31 affected the colon cancer partly in a p53 dependent manner. In serous ovarian carcinomas, miR-31 is underexpressed, and, in a number of serous cancer cell lines with a dysfunctional p53 pathway, miR-31 overexpression inhibits proliferation and induces apoptosis; however, in other lines with functional p53, miR-31 has no effect [18]. Taken together, it suggests that miR-31 may regulate different processes in different cancers, dependent on the cell of origin of the cancer.

The lack of knowledge about the targets for miR-31 in CRC hampers a full understanding on the biological functions of miR-31. Bioinformatic analysis reveals that miR-31 can control the expression of more than one hundred genes (miRBASE, http://microrna.sanger.ac.uk). It may be expected that the targets of miR-31 belong to the class of tumor suppressor genes or genes encoding proteins with potential tumor suppressor functions. BAP1, a tumor suppressor gene that functions in the BRCA1 growth pathway [29]; HIF1AN, inhibits HIF1A transcriptional activity [30]; MAPK transduction protein such as MAP4K5; RAS homologues RAB14, RAB6B, and RASA1 were found as potential targets of miR-31. MiR-31 is also up-regulated in HNSCC and lung cancer. More recently, factor-inhibiting hypoxia-inducible factor (FIH) was experimentally verified as the target of miR-31 in HNSCC cell and the tumor-suppressive genes, large tumor suppressor 2 (LATS2) and PP2A regulatory subunit B alpha isoform (PPP2R2A) were the targets of miR-31 in lung cancer [11,14]. Whether these experimentally verified targets in other cancers are validated in CRC or there are other special targets in CRC need to be answered by further studies.

## Conclusions

Our findings suggest that suppression of miR-31 increases sensitivity to 5-FU at an early stage, and affects cell migration and invasion in HCT-116 colon cancer cells.

## Competing interests

The authors declare that they have no competing interests.

## Authors' contributions

CJW carried out the experiments and drafted the manuscript. JS participated in the experiment and collected the data. ZGZ participated in study design. XFS conceived of the study, participated in the design and helped in the drafting of the manuscript. All authors read and approved the final manuscript.

## Pre-publication history

The pre-publication history for this paper can be accessed here:

http://www.biomedcentral.com/1471-2407/10/616/prepub
